# Supplementary material for: Spiroplasma eriocheiris FtsZ assembles the ring-like structure assisted by SepF
Source: J Biol Chem. 2025 Mar 4;301(4):108373. doi: 10.1016/j.jbc.2025.108373 (PMC11987601; doi:10.1016/j.jbc.2025.108373)
Supplement: Supplemenatry Materials [file mmc1.pdf]

***Spiroplasma eriocheiris* FtsZ assembles the ring-like structure assisted by SepF**

Taishi Kasai[1], Yuhei O. Tahara[2,3], Makoto Miyata[2,3], Daisuke Shiomi[1]*

[1]Department of Life Science, College of Science, Rikkyo University
[2]Graduate School of Science, Osaka Metropolitan University
[3]The OMU Advanced Research Center for Natural Science and Technology, Osaka Metropolitan University

***Corresponding author**
Daisuke Shiomi
Department of Life Science, College of Science, Rikkyo University, 3-34-1 Nishi Ikebukuro, Toshima-ku, Tokyo 171-8501, Japan
Email: dshiomi@rikkyo.ac.jp

Running title: FtsZ of cell wall-less bacteria

Keywords: cell division, FtsZ, SepF, *Spiroplasma*, L-form

```
Bacillus subtilis           ---MSMKNKLKNFFSMEDEE-YEYEYIETERESHEEHEQKEKPAYNGNKP   46
Listeria monocytogenes      ---MGLSNKFKSFFFLDEEEEYYEEEVAREPEPMQKKTKKEKPNKNRFYA   47
Staphylococcus aureus       MSHLALKDLFSGFFVIDDEEEVEVPDKQQQVNEAPAKEQSQQTTKQNAIK   50
Corynebacterium glutamicum  ---MSMLKKTKEFFGLAPYEAEHEDAYYADEPRYEGTAAYAPEYRERDYG   47
Spiroplasma eriocheiris     ---MGIFKKKRSTVVDQPANRIDFSENPHRDNNDLDYEKTNDNIETSN-L   46
Spiroplasma melliferum      ---MSIFKKKQRSIYDAQP-RIDFNDSPTEPIVHSDQPLAKPVLAPETPF   46
Mycoplasma mycoides         ----MFFKKKKNFFKQNEQDQDQIELELTDSDIFEQESPVLKDSYTQTSN   46
                                 : .     .

Bacillus subtilis           AGKQ--------------------------------NVVSLQSVQKSSKV   64
Listeria monocytogenes      VEEDD------------------------------AKVVSMQGAQFSSRM   67
Staphylococcus aureus       SVPQKSASRYTTTSEERNNRMSNYSKNNSRNVVTMNNATPNNASQESSKM  100
Corynebacterium glutamicum  YAPEAPAP----------------------------VAPSPAPRSYQSTI   69
Spiroplasma eriocheiris     NRVEFEEDGN-------------------------PTSPNLPASFDNAEE   71
Spiroplasma melliferum      QKVNFERENE-------------------------IQNSSETSSDTSVLT   71
Mycoplasma mycoides         QFNQNHQTNT-------------------------SNMNQTDMNKSPINT   71
                               :

Bacillus subtilis           VLSEPRVYAEAQEIADHLKNRRAVVVNLQRIQHDQAKRIVDFLSGTVYAI  114
Listeria monocytogenes      VLAEPRVYAEAQELADYLKEYKSVVVNLQRISHDQATRIVDFLSGTVYAL  117
Staphylococcus aureus       CLFEPRVFSDTQDIADELKNRRATLVNLQRIDKVSAKRIIDFLSGTVYAI  150
Corynebacterium glutamicum  VPVELHSFEDAQVIGGAFRDGDAVVFDMSLLSREEARRIVDFAAGLCFAL  119
Spiroplasma eriocheiris     NSIIPTSFHDGARLADQLIKTRHLQVQLTNLSKEERIRLLDFLSGVMYAL  121
Spiroplasma melliferum      TGFIADSYNDAPKMADLLLTKGQLIVHLEKLPKAERIRLLDFISGVMYAF  121
Mycoplasma mycoides         YVFSPMKFSEVQSIVDTLLDQKVVVVDFKNLDDNKAKRFKDFLSGVLYIK  121
                                   : :   : . :       ..:  :   .  *: ** :*  :

Bacillus subtilis           GGDIQRIGSDIFLCTPDNVDVSGTISELISEDEHQRW  151
Listeria monocytogenes      GGDIQRVGNNIFLCTPDNVEVDGSISEMLDEQNFM--  152
Staphylococcus aureus       GGDIQRVGTDIFLCTPDNVEVAGSITDHIENMEHSFD  187
Corynebacterium glutamicum  RGKMQKIDSVTFAVVPELSNISTSELERAARIR----  152
Spiroplasma eriocheiris     NGKVSKLANKTYEFVLQG-------------------  139
Spiroplasma melliferum      DGDVQKIEYKTYQFTIQKDA-----------------  141
Mycoplasma mycoides         KGEYIRLNENIYKFIIN--------------------  138
                             *.  ::    :    :
```

Figure S1. Multiple alignment of SepF from various bacteria.
The alignment was prepared by ClustalW. Asterisk and colon, period indicate conserved residues, residues with strongly similar properties and residues with weakly similar properties, respectively. G109, G116 and G137 of *B. subtilis* SepF are highlighted in green, purple and red, respectively.

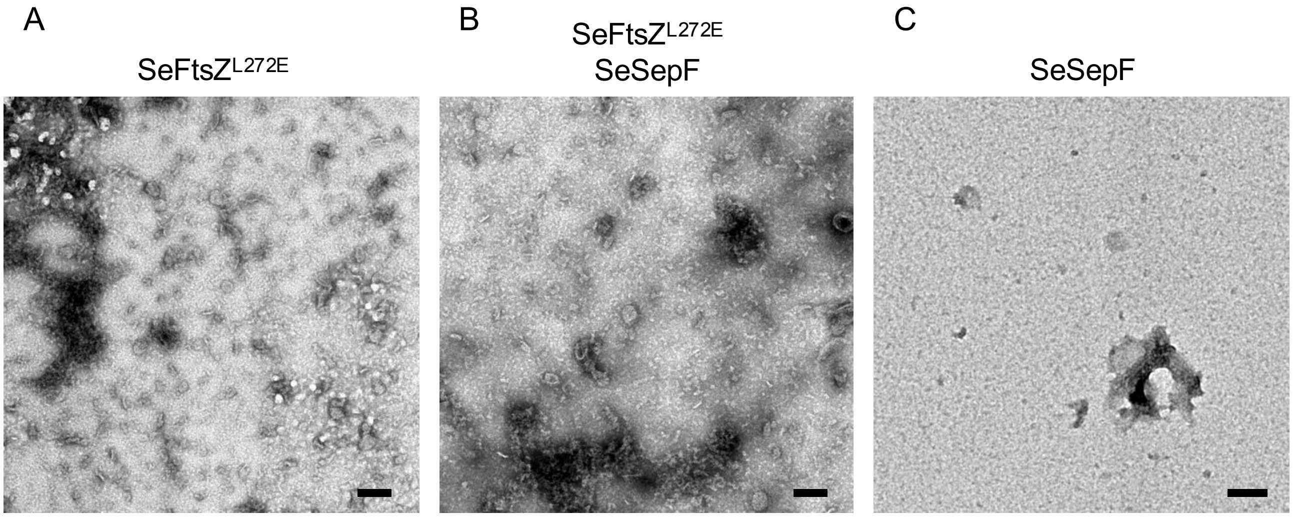

Figure S2. Electron microscope image of SeFtsZ^{L272E} and SeSepF. SeFtsZ^{L272E} did not polymerize without (A) or with (B) SeSepF. (C) SeSepF was not formed the ring-like structure at neutral pH. Scale bar: 100 nm.

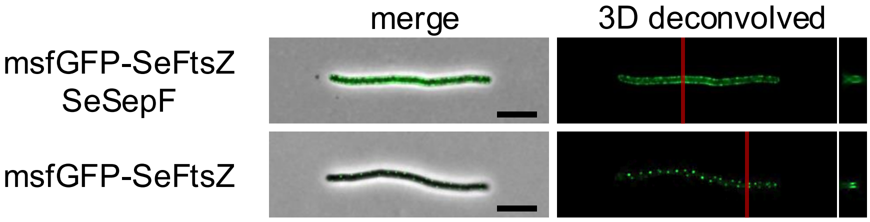

Figure S3. Subcellular localization of SeFtsZ.
The three-dimensional deconvolved images of msfGFP-SeFtsZ focus and ring with (top) or without (bottom) SeSepF. A cross-section of a red line is shown. Scale bar: 2 μm.

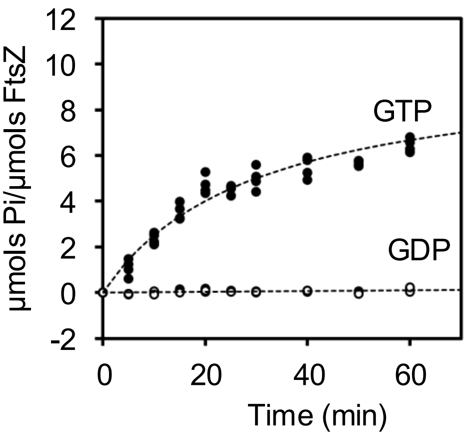

Figure S4. GTP hydrolysis during SeFtsZ^{F226M} mutant polymerization. SeFtsZ^{F226M} (12 µM) polymerized with 1 mM GTP or GDP.
